# Matrix metalloproteinase-8: a useful biomarker to refine the diagnosis of community-acquired pneumonia upon intensive care unit admission?

**DOI:** 10.1186/s13054-019-2513-7

**Published:** 2019-06-20

**Authors:** Fabrice Uhel, Brendon P. Scicluna, Lonneke A. van Vught, Olaf L. Cremer, Marc J. Bonten, Marcus J. Schultz, Tom van der Poll

**Affiliations:** 1 Center of Infection and Immunity Amsterdam (CINIMA), Amsterdam University Medical Centers, location Academic Medical Center, University of Amsterdam, Amsterdam, The Netherlands; 2 Center for Experimental and Molecular Medicine, Amsterdam University Medical Centers, location Academic Medical Center, University of Amsterdam, Amsterdam, The Netherlands; 3 Department of Intensive Care Medicine, University Medical Center Utrecht, Utrecht, the Netherlands; 4 Department of Medical Microbiology, University Medical Center Utrecht, Utrecht, the Netherlands; 5 Julius Center for Health Sciences and Primary Care, University Medical Center Utrecht, Utrecht, the Netherlands; 6 Department of Intensive Care Medicine, Amsterdam University Medical Centers, location Academic Medical Center, University of Amsterdam, Amsterdam, The Netherlands; 7 Division of Infectious Diseases, Amsterdam University Medical Centers, location Academic Medical Center, University of Amsterdam, Amsterdam, The Netherlands

**Keywords:** Sepsis, Pneumonia, Biomarker, Matrix metalloproteinase-8, Procalcitonin, Infection

The optimal management of severe community-acquired pneumonia (CAP) requires a prompt and accurate diagnosis [1]. Since clinical, radiological, and biological findings are poorly sensitive or specific, microbiological documentation often slow and unavailing, biomarkers could help to safely withhold antibiotics when the risk of bacterial infection is minimal and steer the diagnostic process towards non-infectious causes of respiratory failure [2]. In our previous study deriving the *FAIM3:PLAC8* molecular biomarker, we noticed that *MMP8*, encoding matrix metalloproteinase-8 (MMP-8), was the most overexpressed gene in confirmed CAP relative to non-infectious differential diagnoses (no-CAP) [3]. We investigated in the same cohort if plasma levels of MMP-8 could be a valuable candidate biomarker for the diagnosis of CAP.

Similar to *MMP8* whole blood gene expression (Fig. 1a), plasma MMP-8 (measured by Luminex assay [BioRad, Hercules, CA, USA]) was increased in patients with a suspicion of CAP compared to healthy volunteers and further increased in patients with confirmed CAP (median 3.45 ng/mL; interquartile range [IQR], 0.93–15.40 ng/mL, *n* = 86) compared to no-CAP (0.76 ng/mL; IQR, 0.35–2.64 ng/mL, *p* < 0.001, *n* = 31, Fig. 1b). *MMP8* expression correlated with plasma levels of MMP-8 (rho = 0.73, *p* < 0.001, Fig. 1c). The receiver operating characteristic area under the curve (AUC) of plasma MMP-8 for the prediction of infection was 0.71 (95% CI 0.59–0.81) (Fig. 1d). A numerical threshold set at 0.25 ng/mL to minimize the risk of false-negative diagnosis allowed the identification of infection with a 97% sensitivity at the expense of a low specificity (19%). AUCs for plasma MMP-8, *MMP8* expression, and procalcitonin (widely used for the diagnosis of CAP [4]) were not statistically different (Fig. 1d). In the independent validation cohort comprising 57 CAP and 26 no-CAP patients, the AUC for MMP-8 was 0.83 (95% CI 0.73–0.91, Fig. 1e). A numerical threshold of 0.30 ng/mL favoring a > 97% sensitivity yielded a specificity of 15%. The combination of MMP-8 (cutoff 0.25 ng/mL) with a reference model including variables routinely used for the diagnosis of infection (body temperature ≥ 37.5 °C and procalcitonin > 1.0 ng/mL [5]) significantly but modestly improved the prediction of infection (net reclassification improvement 0.36 [95% CI 0.03–0.70], *p* = 0.033).

**Fig. 1:**
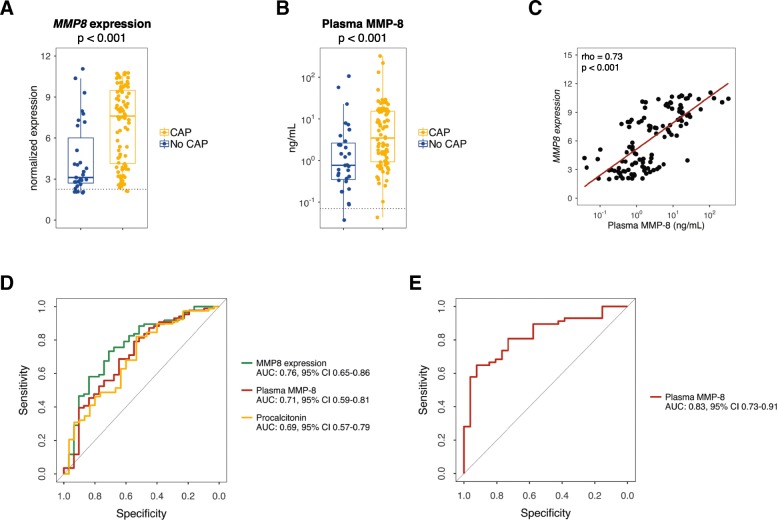
Comparison of *MMP8* expression, MMP-8 plasma levels, and procalcitonin in consecutively enrolled patients treated for suspected community-acquired pneumonia (CAP) upon intensive care unit admission. **a** Box-and-whisker and dot plots depicting *MMP8* expression in CAP (*n* = 86) and no-CAP patients (non-infectious control, *n* = 31). The dotted line represents *MMP8* median expression in age-matched healthy volunteers (*n* = 42). **b** Box-and-whisker and dot plots depicting MMP-8 plasma levels in CAP and no-CAP patients. The dotted lines indicate median values obtained in 27 age-matched healthy subjects. **c** Correlation between *MMP8* expression and MMP8 plasma levels in patients admitted for a suspected CAP. **d** Comparison of *MMP8* expression or plasma levels with procalcitonin in patients consecutively admitted to the ICU for a suspicion of CAP (cohort A). Receiver operating characteristic analysis. AUC, area under the curve. **e** Assessment of the MMP8 plasma biomarker in an independent cohort (Validation cohort) of CAP (*n* = 57) and no-CAP patients (*n* = 26). Receiver operating characteristic analysis AUC

In conclusion, MMP-8 slightly improved patient classification compared to a routine care reference strategy. However, its poor specificity precludes its use as a stand-alone diagnostic biomarker to safely withhold antibiotics in this critically ill population. Further studies are needed to establish the potential add-on value of plasma MMP-8 in diagnostic tests including multiple biomarkers.

## Data Availability

Gene expression datasets are available at the Gene Expression Omnibus public repository of NCBI under accession number GSE65682. Other data generated and/or analyzed during the current study are available from the corresponding author on reasonable request.

## Abbreviations

AUC: Area under the curve
CAP: Community-acquired pneumonia
IQR: Interquartile range
MMP-8: Matrix metalloproteinase-8
ROC: Receiver operating characteristic
